# Subcutaneous Ligature of an Artery

**Published:** 1892-07

**Authors:** Thomas. J. Pugh

**Affiliations:** Hearne, Texas


### DANIEL'S TEXAS MEDICAL JOURNAL.

ESTABLISHED JULY, 1885.

PUBLISHED MONTHLY.—SUBSCRIPTION $2.00 A YEAR.

Vol. VIII. AUSTIN, JULY, 1892. No. 1.

### Original Contributions.

For Daniel's Texas Medical Journal:

### SUBCUTANEOUS LIGATURE OF AN ARTERY.

BY THOMAS. J. PUGH, M. D., HEARNE, TEXAS.

LIGATION after cutting down on an artery and deligation by compression, is a practice that falls to the lot of every surgeon. But so far as I know, subcutaneous ligation, save in operations for varicocele is a practice unknown and not practiced by our surgeons of to-day.

In December, 1884, I amputated the arm of a negro, just below the elbow. This man was caught in the moving machinery of a steam gin, on the Cavitt plantation, in Brazos county, Texas. All the metacarpal bones of the hand were broken. The ulna and radius were broken at the middle and lower third, and the bones were split up and protruded through the bruised and lacerated flesh. The os humeri was also broken above the elbow.

Amputation being called for, I did the operation at the upper third of the forearm, and put the broken os humuri in pasteboard splints and bandage. After tying the principal arteries—the radial and ulnar—I found there was another bleeding vessel which I could not reach and tie, and in my dilemma I concluded to use subcutaneous ligation. Acting upon this idea, I simply passed a curved needle into and around the bleeding mass, and tied the same. The bleeding was immediately controlled, and the man, without much pain, recovered entirely.

I suppose almost any other surgeon would have amputated this man's arm above the elbow, at the site of the break in the os humeri.

I claim some special credit for saving the broken arm by setting and at the same time having to amputate the same member below the elbow.

On the 15th of June, 1892, I was called in consultation with Drs. Lewis and Erwin, of Hearne, to see one Taylor Whitehead, who six days before had cut a large, long, deep gash in his foot, severing the dorsalis pedis artery. Local astringents and compression had been used for six days and nights, with no good results. It was suggested that we cut down on the anterior tibial artery at the lower third, and tie the artery. I objected, as I thought cutting was unnecessary. The doctors left the matter to me, and after having Dr. Erwin chloroform the patient, I proceeded to pass a large curved needle armed with strong silk around the artery, and out on the opposite side from entrance. This of course included the skin, muscles, nerves and blood vessels. The hemorrhage ceased at once. The stitches were withdrawn in four days, and the patient is now up and about the house, and will recover.

The idea I wish to convey to the profession is the non-necessity of cutting down on an artery. Even in an aneurism, if the artery can be reached with a needle and thread through the skin and muscles, why not pass the ligature subcutaneously around the artery, instead of cutting down to the vessel? On account of pain produced by including the nerve in the ligature, some might object to this procedure, but no great pain follows the ligation of the nerve with the other tissues. An occasional dose of morphine will insure against pain, and will produce rest. In my opinion, subcutaneous ligation should be resorted to in all cases where the artery can be reached without cutting.

